# Dopaminergic neurons: linking longevity with reproduction?

**DOI:** 10.18632/aging.204000

**Published:** 2022-04-02

**Authors:** Xiaolin Tian

**Affiliations:** 1 Neuroscience Center of Excellence, Department of Cell Biology and Anatomy, Louisiana State University Health Sciences Center, New Orleans, LA 70112, USA

**Keywords:** dopaminergic neurons, aging, reproduction, longevity, aging theory

Dopaminergic neurons are critical modulators for essential brain functions such as learning and memory, reward and addiction, motor control, and metabolism. My recent work identified a novel function of dopaminergic neurons in regulating aging and longevity in flies [1]. I demonstrated that overexpressing the putative scaffolding protein Mask in small subsets of dopaminergic neurons significantly extends the lifespan in flies. Interestingly, the prolonged lifespan of the Mask-overexpressing flies is accompanied by sustained reproductive activities, contradicting the long-acknowledged inverse relation between reproduction and longevity. This prevalent negative correlation between reproduction and longevity has been explained by the disposable soma theory that posits a competing allocation of energy between reproduction and somatic maintenance. However, my work, together with a few other findings in flies [2–5], suggested that extension of both lifespan and reproduction can be induced simultaneously by a variety of specific genetic manipulations. Moreover, such a co-extension also occurs in nature – the reproductive females of eusocial insects acquire physiological transformations that enable the expansion of both their reproduction capacity and lifespan. It seems that a common mechanism may exist to actively induce adaptations in the soma to cope with the reproductive demands of the animals, which also at the same time intervenes the aging process and extends the lifespan. Inspired by this notion, I propose a reproduction-centered adaptable soma theory that consensually explains the seemingly contradictory relationships of reproduction and longevity. The success of reproduction is essential for the survival of the species. From such a reproduction-centered perspective, the maintenance of the somatic tissues is not just critical for the survival of individuals but is, more importantly, essential for the fulfillment of reproduction. Therefore, I postulate that somatic tissues possess the ability to adapt to, instead of competing with, the animal’s reproduction states and that such adaptations can consequentially impact aging and longevity. For example, the scarcity of nutrients might serve as signals for insufficient resources needed to sustain the production of quality progenies, which, in turn, leads to an adaptive strategy that prolongs lifespan and delays reproduction so that the reproduction could be resumed later when the condition improves. Indeed, starved male flies actively suppress their mating drive but can quickly transit from feeding to courtship when food is available [6]. In eusocial insects, the somatic adaptation takes place naturally – the reproductive females undergo somatic remodeling so that they can live much longer to meet the reproduction demand. On the other hand, fast reproduction in early life correlates with shorter lifespan, which may reflect the lift of somatic maintenance resulted from the early fulfillment of the reproduction.

The greatest hurdle toward testing and proving this new theory for aging and reproduction is the lack of knowledge about the system and mechanisms that coordinate somatic maintenance with reproductive status. It is not clear whether the somatic and reproductive tissues interact in a linear hierarchical order or they mutually act on each other. It is also unknown how the reproductive status is expressed, sensed, processed, and incorporated into a “decision” for execution of somatic adaptation, i.e. to alter the status of the somatic maintenance accordingly.

The identification of specific dopaminergic neurons as potent regulators for both the life span and the reproduction span may provide a good entry point to explore the mechanisms of a reproduction-driven somatic adaptation. The dopaminergic system, capable of facilitating information evaluation and decision-making, is a suitable candidate to coordinate not only the processing of sensory inputs related to the nutritional and the reproductive status but also the commanding of physiological adjustment in the somatic tissues. First, the dopaminergic system is part of the neuronal-driven mechanism for mating and reproduction behaviors that control ovarian dormancy in female flies and regulate sex activities in male animals [7]. The dopaminergic system also modulates the sensory aspects of mating, such as the sensitivities of the auditory systems in the midshipmen fishes during seasonal mating. Second, dopaminergic neurons regulate energy balance and modulate systemic physiological functions [8]. Thus, the dopaminergic system possesses the characteristics required for serving as a central element of the mechanism that coordinates the somatic adaptation and reproductive states. Future studies aimed to identify the dopaminergic circuits in the central and peripheral nervous system and their downstream effector neurons and somatic tissues will bring insight into the molecular and cellular mechanisms associated with the new adaptable soma theory for aging and reproduction.
